# T183. AUDITORY-STEADY-STATE RESPONSES AND CORTICAL VOLUME IN PATIENTS WITH SCHIZOPHRENIA

**DOI:** 10.1093/schbul/sby016.459

**Published:** 2018-04-01

**Authors:** Do-Won Kim, Sungkean Kim, Seon-Kyeong Jang, Jihoon Kang, Chang-Hwan Im, Seung-Hwan Lee

**Affiliations:** 1 Chonnam National University; 2 Hanyang University; 3 Clinical Emotion and Cognition Research Laboratory; 4 Clinical Emotion and Cognition Research Laboratory, Inje University IIsan Paik Hospital

## Abstract

**Background:**

The 40-Hz auditory steady-state response (ASSR) probing gamma-band oscillations may reflect N-methyl-D-aspartate receptor (NMDAR) dysfunction in patients with schizophrenia (SZ). Diminished gamma oscillations are reported in SZ, although increased spontaneous gamma oscillations are also reported. We investigated the 40-Hz ASSR and its association with brain volumes and clinical symptoms of SZ.

**Methods:**

The 40-Hz ASSR was measured using electroencephalography in 33 patients with SZ and 30 healthy controls (HCs). Four gamma oscillation components (evoked power, spontaneous oscillations (baseline and total power), and inter-trial phase coherence (ITC)) were assessed. Brain volumes were assessed using high-resolution magnetic resonance imaging and voxel-based morphometry.

**Results:**

Patients with SZ had larger evoked and total powers and higher ITC than HCs. In HCs, evoked power showed significant positive correlations with bilateral superior temporal gyrus (STG) volume. In SZ, the effect of positive symptoms on the path from evoked power to left STG volume was significantly moderated. In SZ with elevated positive symptoms, large evoked power predicted small left STG volume, whereas large evoked power predicted large left STG volume in those with low positive symptoms. Increased baseline power was associated with a smaller left middle frontal gyrus (MFG) volume in SZ, whereas increased ITC correlated with larger MFG volume in HCs.

**Discussion:**

Our results support the NMDAR hypofunction model of SZ, and suggest significant involvement of the STG and MFG in gamma oscillations.

